# Effect of multiple micronutrient supplementation during pregnancy on maternal and birth outcomes

**DOI:** 10.1186/1471-2458-11-S3-S19

**Published:** 2011-04-13

**Authors:** Batool Azra Haider, Mohammad Yawar Yakoob, Zulfiqar A Bhutta

**Affiliations:** 1 Division of Women & Child Health, The Aga Khan University, Karachi, Pakistan

## Abstract

**Objectives/background:**

Given the widespread prevalence of micronutrient deficiencies in developing countries, supplementation with multiple micronutrients rather than iron-folate alone, could be of potential benefit to the mother and the fetus. These benefits could relate to prevention of maternal complications and reduction in other adverse pregnancy outcomes such as small-for-gestational age (SGA) births, low birth weight, stillbirths, perinatal and neonatal mortality. This review evaluates the evidence of the impact of multiple micronutrient supplements during pregnancy, in comparison with standard iron-folate supplements, on specific maternal and pregnancy outcomes of relevance to the Lives Saved Tool (LiST).

**Data sources/review methods:**

A systematic review of randomized controlled trials was conducted. Search engines used were PubMed, the Cochrane Library, the WHO regional databases and hand search of bibliographies. A standardized data abstraction and Child Health Epidemiology Reference (CHERG) adaptation of the Grading of Recommendations Assessment, Development and Evaluation (GRADE) technique were used for data abstraction and overall quality of evidence. Meta-analyses were performed to calculate summary estimates of utility to the LiST model for the specified outcome of incidence of SGA births. We also evaluated the potential impact of multiple micronutrients on neonatal mortality according to the proportion of deliveries occurring in facilities (using a threshold of 60% to indicate functionality of health systems for skilled births).

**Results:**

We included 17 studies for detailed data abstraction. There was no significant benefit of multiple micronutrients as compared to iron folate on maternal anemia in third trimester [Relative risk (RR) = 1.03; 95% confidence interval (CI): 0.87 – 1.22 (random model)]. Our analysis, however, showed a significant reduction in SGA by 9% [RR = 0.91; 95% CI: 0.86 – 0.96 (fixed model)]. In the fixed model, the SGA outcome remained significant only in women with mean body mass index (BMI) ≥ 22 kg/m^2^. There was an increased risk of neonatal mortality in studies with majority of births at home [RR = 1.47, 95% CI: 1.13-1.92]; such an effect was not evident where ≥ 60% of births occurred in facility settings [RR = 0.94, 95% CI: 0.81-1.09]. Overall there was no increase in the risk of neonatal mortality [RR = 1.05, 95% CI: 0.92 – 1.19 (fixed model)].

**Conclusion:**

This review provides evidence of a significant benefit of MMN supplementation during pregnancy on reducing SGA births as compared to iron-folate, with no significant increase in the risk of neonatal mortality in populations where skilled birth care is available and majority of births take place in facilities. Given comparability of impacts on maternal anemia, the decision to replace iron-folate with multiple micronutrients during pregnancy may be taken in the context of available services in health systems and birth outcomes monitored.

## Introduction

Low birth weight, small-for-gestational age (SGA), preterm birth, stillbirths, perinatal and neonatal mortality are important adverse outcomes of pregnancy [1]. The incidence of low birth weight in developing countries varies from 6 - 30%, and at least one-third of these are small for gestational age, especially in settings with high rates of maternal undernutrition. Small for gestational age (SGA) babies are those whose birth weight lies below the 10^th^ percentile for a particular gestational age [2]. Vast majority of these are due to fetal growth problems that occur during pregnancy, including intrauterine growth restriction (IUGR) [3]. Full term SGA infants may not have complications related to organ immaturity like those of pre-term infants of similar size, but are at an increased risk of stillbirth and perinatal/neonatal mortality due to perinatal asphyxia, meconium aspiration and hypoglycaemia [4,5]. Women of reproductive age, especially pregnant women, in developing countries are recognized to be at risk of multiple micronutrient deficiencies, such as iron, folic acid, iodine, zinc, vitamins A and D, riboflavin, B6 and B12, with the likelihood of adverse effects on the mother and pregnancy outcomes [1,6]. Pregnancy represents a state of increased metabolic requirements, and intake of key micronutrients by pregnant women especially in developing countries is usually inadequate. This inadequate intake and increased requirement further exacerbates the pre-existing maternal deficiency [7].

Iron deficiency contributes to one of the largest prevalence of micronutrient deficiencies among pregnant women. For example, anaemia affects approximately 41.8% of all pregnancies globally [8], with iron deficiency accounting for half of the cases [9]. Retrospective and observational studies have demonstrated a higher risk of maternal mortality in severely anaemic pregnant women, predisposing to death from haemorrhage and infections. Maternal iron deficiency anaemia also has adverse effects on birth outcomes including a greater risk of birth asphyxia, low birth weight, preterm delivery and lower Apgar scores [10,11]. The association of maternal anemia with adverse outcomes is largely based on observational studies and risk assessment [12] and objective evidence that iron-folate supplementation in pregnancy improves outcomes other than anemia is not well established.

Given the significant impact of deficiencies of key micronutrients during pregnancy [6]**,** supplementation with multiple micronutrients during pregnancy may be a feasible public health strategy. One potential advantage of multiple micronutrients could be that they might have comparable benefits to iron-folate in reducing anemia, and could also have additional benefits on intrauterine growth and outcomes in the neonatal period and infancy [7]. Many workers had attempted augmentation of iron-folate supplementation in pregnancy with additional micronutrients, but the first systematic efforts to undertake this were almost a decade ago [7]. In 1999 the UNICEF/WHO/UN University proposed a prenatal supplement UNIMAPP containing fifteen micronutrients, including iron and folic acid which could provide one recommended daily allowance of each and potentially replace standard iron-folate supplements for pregnant women in low and middle income countries [13]. It was speculated that a combination of more than one micronutrient may have additive and/or synergistic benefit on maternal and child outcomes. Systematic information on the benefits if any, of such supplements in improving maternal and pregnancy outcomes is, however, limited. A Cochrane review on the subject in 2006 [14] indicated that compared to iron-folate, multiple micronutrient supplementation (defined as administration of three or more micronutrients), did not have any significant benefits on maternal anemia (data from one study) and prevalence of SGA births (data included from 2 studies). Another systematic review was undertaken by a team commissioned by UNICEF/WHO/SCN which analysed data from 12 trials using the UNIMAPP formulation and evaluated effects on maternal and pregnancy outcomes [15] There have been concerns regarding increased risk of perinatal and neonatal mortality with multiple micronutrient supplementation through increased birth asphyxia in heavier babies [9] In this paper, we included additional studies since the Cochrane review in 2006 and have evaluated the evidence of the potential impact of multiple micronutrient supplementation in pregnancy on the maternal and pregnancy outcomes using Child Health Epidemiology Reference Group (CHERG) rules [16]. Given that the Lives Saved Tool (LiST) largely models SGA outcomes for potential maternal nutrition interventions, we specifically focused our analysis to evaluate the impact of maternal multiple micronutrient interventions on risk of delivering SGA infants and also evaluated the impact on neonatal mortality.

## Methods

### Searching

We evaluated all relevant literature on maternal multiple micronutrient supplementation during pregnancy for assessment of effects on pregnancy outcomes, up to December 2009. The databases searched included PubMed, the Cochrane Systematic Reviews, the World Health Organization Regional Databases and hand search of bibliographies of relevant reviews. Experts were also contacted in the field for unpublished data. The basic search strategy used was:

("Mothers"[Mesh] OR "Pregnancy"[Mesh] OR mother* OR maternal OR pregnancy) AND ("Micronutrients"[Mesh] OR "multiple micronutrient*" OR multivitamin OR micronutrient*) AND (supplement*)

### Selection (inclusion/exclusion criteria)

All prospective randomized controlled trials (RCTs) evaluating multiple micronutrient supplementation in women during pregnancy, irrespective of language or publication status, were included. Multiple micronutrients were defined as supplementation with at least 5 micronutrients including the UNIMMAP formulation [13] or those with comparable composition. These supplements were compared to maternal iron-folate supplementation. There were no limits on gestational age at the time of enrolment in the study and the duration of supplementation. Quasi-randomized trials were excluded as there was an adequate number of good quality RCTs available. We did not conduct sub-group analyses with respect to different dosages of iron in the multiple micronutrient supplements. Other than the assessment of SGA and neonatal mortality, we did not specifically evaluate minor adverse effects of the supplements such as nausea and vomiting among the mothers and newborns.

### Validity assessment

The overall quality of evidence of an outcome, however, was assessed and graded according to the CHERG adaptation of the Grading of Recommendations Assessment, Development and Evaluation (GRADE) technique [16,17] based on three components: 1) the volume and consistency of the evidence; 2) the size of the effect, or risk ratio; and 3) the strength of the statistical evidence for an association between the intervention and outcome, as reflected by the p-value [16]. The individual studies were also graded. Three categories of criteria were used to judge quality of individual study evidence in the meta-analysis: 1) study design; 2) study quality; 3) relevance to the objectives of the review [16]. The following four grades were given to individual studies: high, moderate, low or very low. Study received an initial score of high if it was a randomized or cluster randomized trial. The grade was decreased by 0.5 to 1 for each study design limitation. In addition, studies reporting an intent-to-treat analysis or with statistically significant strong levels of association (>80% reduction) received 0.5-1 grade increases. Any study with a final grade of very low was excluded on the basis of inadequate study quality. This review is shaped in large part by the needs of the LiST model. In that model, increases in coverage of an intervention result in a reduction of one or more cause-specific deaths or in reduction of a risk factor. Therefore, this review and the grade process used are designed to develop estimates of the effect of an intervention in reducing either a risk factor or a death due to specific cause. For more details of the review methods, the adapted grade approach or the LiST model, see the CHERG method’s paper [16]. For the LiST tool, we have defined SGA as an outcome rather than low birth weight as the model utilizes the former for the cohort effect. The SGA babies, belonging to the least 10^th^ centile of the birth weight, would be at a higher risk of mortality and thus a greater effect of any intervention. Besides, in several populations used in the studies, the two terms have been used synonymously.

### Data abstraction and study characteristics

Each study that satisfied the eligibility criteria was included in the review. Data were double abstracted into a standardized rectangular database [16] that was accessible through Excel (Additional File 1). Key variables like participants’ characteristics, sample size, location, setting, blinding, allocation concealment, description of intervention and control groups (in terms of dosage and time of enrolment) and all the other outcomes of interest were recorded.

### Quantitative data synthesis

The assessment of statistical heterogeneity among trials was done by visual inspection i.e. the overlap of the confidence intervals among the studies, and by the Chi square (P-value) of heterogeneity in the meta-analyses. A low P value (less than 0.10) or a large chi-squared statistic relative to its degree of freedom was considered as providing evidence of heterogeneity. The I^2^ values were also looked into, and roughly an I^2^ greater than 50% was taken to represent substantial and high heterogeneity. In situations of substantial or high heterogeneity being present, causes were explored, sub-group analyses performed and random effects model was used and although, this random model is not a substitute for a thorough investigation of heterogeneity, it was primarily to take into account heterogeneity that could not be explained [18]. Pooled estimates were generated by generic inverse variance method of meta-analysis using Review Manager (version 5) software. For all cluster randomized trials, cluster adjusted estimates were used. For the purpose of the analyses of the studies with factorial designs, we have assumed that there was no interaction between other interventions and the effect of supplementation, since all interventions were randomized. Results are presented as risk ratios (RR) and corresponding 95% confidence intervals (CI). We summarized evidence of the outcomes including qualitative grading of the study quality and quantitative attributes according to the reference guidelines [16]. The CHERG Rules for Evidence Review were applied to the outcomes of SGA and neonatal mortality. For SGA, sub-group analyses were performed according to the mean maternal body mass index (BMI). In an effort to understand the context of neonatal outcomes, we evaluated the effect of multiple micronutrient supplements on neonatal mortality by sub-group analysis according to the percentage of facility based deliveries. Not all studies provided sufficient data to allow categorization of health system functionality for maternal health, but information on facility births or home births was available. We used an arbitrary cut off of 60%, where more than 60% facility births represented a proxy for skilled attendance.

## Results

### Trial flow

**Figure 1:**
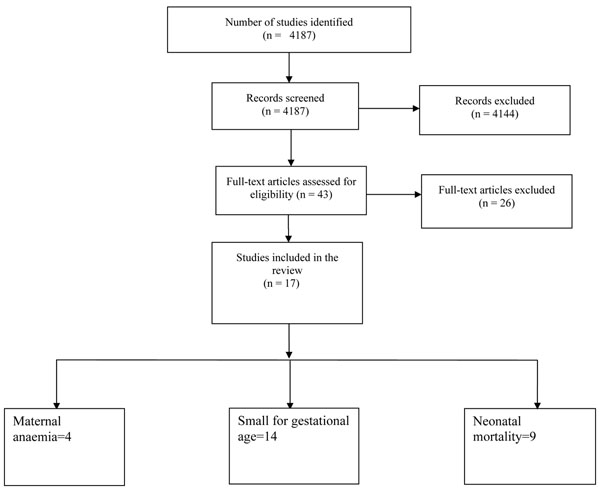
Flow diagram showing identification of studies

A total of 4,187 hits were identified from our search strategy (Figure 1). After screening the titles and abstracts, 43 studies were initially considered eligible and finally, 17 studies comprising of 14 trials were selected for inclusion in this review. We evaluated the impact of the intervention on the following outcomes: maternal anemia, SGA and neonatal and early infant mortality.

### Study characteristics

The baseline characteristics of all the studies including the location, sample size, type of participants, intervention composition and limitations are given in Additional File 2. Six out of fourteen included studies had used cluster randomized design [19-26]. All studies were from low-/middle-income settings. Some of the studies had limitations based on study design and execution such as large losses to follow-up, insufficient power to detect differences in SGA and mortality, and missing compliance data. Twelve studies used multiple micronutrient supplement formula called UNIMMAP which consisted of 30 mg iron, 400 µg folic acid, 15 mg zinc, 2 mg copper, 65 µg selenium, 800 µg RE vitamin A, 1.4 mg vitamin B1, 1.4 mg vitamin B2, 18 mg niacin, 1.9 mg vitamin B6, 2.6 µg vitamin B12, 70 mg vitamin C, 5 µg vitamin D, 10 mg vitamin E and 150 µg iodine. The formulas used in rest of the studies were comparable to UNIMMAP except in small variation in dose of iron and folic acid used. Additional File 3 presents the risk of bias in all studies in accordance with the latest recommendations of the Cochrane Handbook [18].

### Quantitative data synthesis

Table 1 reports the overall quality grading of the outcomes and results of the corresponding meta-analyses. The minimum number of micronutrients in the studies was nine. In most studies, the comparison (control) supplement was composed of 60 mg of iron and 400 µg of folic acid. The UNIMAPP formulation containing 30 mg iron and 0.4 mg folic acid was provided in nine trials [19-22,24,27-30]. In study by Friis et al. [31], iron-folate was not part of the intervention composition and iron-folate tablets were provided separately as part of routine antenatal care. The intervention group in the Fawzi et al. study [32] received multiple micronutrient tablets containing 0.8 mg of folic acid along with separate iron and folic acid supplementation. In the study by Gupta et al. [33], the multi-micronutrient tablet contained 10 mg of ferrous fumarate and 0.15 mg of folic acid along with supplemental iron-folate. The composition of intervention in the study by Christian et al. study [23,25,26] included 60 mg iron and 0.4 mg folic acid, while Ramakrishnan et al. [34,35] provided 62.4 mg iron and 0.215 mg folic acid in the multi-micronutrient formulation.

Multiple micronutrient supplementation had no significant effect on maternal anemia in the third trimester compared to iron-folate based on data from 4 RCTs (RR = 1.03; 95% CI: 0.87 – 1.22; random model) [22,25,28,35] (Additional File 4). There was a statistically significant 9% reduction in the risk of small for gestational age infants with multiple micronutrient supplements (RR = 0.91; 95% CI: 0.86 – 0.96; fixed model) based on fourteen studies [19-24,27-34] (Additional File 5A). These results also remained significant with the random effects model (RR = 0.88; 95% CI: 0.81 – 0.95) (Additional File 5B). The sub-group analysis used a cut-off of mean maternal BMI of 22 kg/m^2^. The intervention effect relative to the control group in the fixed model was significant only for women with mean BMI greater than or equal to 22 kg/m^2^ (RR = 0.89, 95% CI: 0.83 – 0.95), but significant for both the sub-groups in the random model.

**Table 1:** Quality assessment of trials of multiple micronutrient supplementation for maternal and pregnancy outcomes (Multiple micronutrients versus iron folate)

	Quality Assessment					Summary of Findings		
				**Directness**		**No of events**		

**No of studies (ref)**	**Design**	**Limitations**	**Consistency**	**Generalizability to population of interest**	**Generalizability to intervention of interest**	**Intervention**	**Control**	**Relative Risk (95% CI)**

***Maternal Anaemia: High*** **outcome specific quality**								

4	cRCT/RCT	Some – high loss to follow-up and inadequate power (-0.5)	Heterogeneity in the meta-analysis = 50%. 2/4 studies showing benefit (-0.5)	All in developing countries	Generalizable	351	336	1.03 (0.87 - 1.22), random model ^a^

***Small-for-gestational age: High*** **outcome specific quality**								

14	cRCT/RCT	Some – high loss to follow-up, inadequate power, missing data (compliance), one study with a co-intervention (-0.5)	Consistent with 13/14 studies showing benefit	All in developing countries	Generalizable	2289	2494	0.91 (0.86, 0.96), fixed model ^a^

***Neonatal mortality:*** ***High*** **outcome specific quality**								

9	cRCT/RCT	Some - inadequate power of the studies (-0.5)	7/9 studies giving a risk ratio of greater than 1	All in developing countries	Generalizable	558	513	1.05 (0.92, 1.19), fixed model ^a^

a = generic inverse variance

Our pooled estimates did not show a statistically significant increase in the risk of neonatal mortality based on nine RCTs [19,21,22,24,26-30] in both the fixed (RR = 1.05; 95% CI: 0.92 – 1.19) (Additional File 6A) and random effect models (RR = 1.17; 95% CI: 0.95 – 1.44) (Additional File 6B). We also performed a sub-group analysis for neonatal mortality with respect to the percentage of facility-based deliveries. This was determined from the information provided in the study, or from the general information on the study location. There was an increased risk of neonatal mortality in studies with majority of births at home [RR 1.47, 95% CI 1.13-1.92]; such an effect was not evident where ≥ 60% of births occurred in facility settings [RR 0.94, 95% CI 0.81-1.09].

## Discussion

Our evaluation of multiple micronutrient supplements during pregnancy did not show a significant benefit of the supplement on maternal anaemia in third trimester as compared to iron-folate. These findings corroborates those from earlier reviews [14,36] which also revealed no significant benefit of multiple micronutrients over iron-folate. However, our review does indicate that multiple micronutrient administration is associated with a significant reduction in SGA births in comparison with iron-folate administration (9% and 12% reduction in the fixed and random effects models, respectively). These findings are comparable to the results of the systematic review of UNIMAPP trials [37] (pooled OR = 0.90; 95% CI: 0.82 – 0.99) based on 12 studies, which are also included in our analysis. These findings are however, different from our previous Cochrane review which was based on a limited set of studies [14] and reported a non-significant effect on SGA (RR = 1.04; 95% CI: 0.93 – 1.17). Our current data are also at variance with a recent meta-analysis by Shah et al. [38] which analyzed results from only 5 RCTs and reported a statistically insignificant impact on SGA (RR = 0.89; 95% CI: 0.77 – 1.01). In our current review, 13 out of 14 studies showed consistent results with respect to SGA babies i.e. a risk ratio of less than 1. The study by Christian et al. reported a risk ratio of greater than 1, but was statistically not significant (RR = 1.04; 95% CI: 0.94 – 1.15) [23]. The overall quality of evidence for this outcome was high, with a strong statistical association with the P-value of 0.0002 in the fixed model. Given that the LiST model uses the impact pathway via SGA, we are recommending a point estimate of 9% reduction in SGA for inclusion in the LiST tool for situations where countries may decide to replace routinely used iron-folate supplements during pregnancy with multiple micronutrients. The sub-group analysis with respect to maternal BMI showed that there was a significant reduction in SGA only in mothers whose mean baseline BMI was ≥ 22 kg/m^2^, and the result was non-significant for BMI less than this. It is difficult to explain these findings as it may in part be an artefact caused by the way mothers’ BMI status were determined. It should to be noted that weight gain in pregnancy is not linear, and women who entered early in the study would be classified as thin compared to those who entered the study in the later stages. Another possible explanations could be hypothesized like decreased ability of malnourished mother to utilize multiple nutrients [39]. However a trial conducted in India on malnourished women did not support this hypothesis [33]. Certain other factors that need to be considered are the age of the mother, composition of multiple micronutrient supplement and co-supplementation of macronutrient like balanced protein energy supplementation [40].

There is considerable debate on the potential adverse effects of providing multiple micronutrient supplements during pregnancy with a concern about an increase in neonatal mortality in less developed health systems that have suboptimal maternal care [41,42]. There are a few studies that reported mortality beyond the neonatal period with maternal multiple micronutrient supplementation, the data of which has not been pooled. Christian et al. [26] had reported infant deaths (0 – 3 months) in the multiple micronutrient recipients versus controls (receiving vitamin A only) with a non-significant higher risk in the micronutrient group (RR = 1.07; 95% CI: 0.75 – 1.58). In contrast, Shankar et al. [21] in the considerably larger SUMMIT trial, reported a statistically significant 18% reduction in early infant mortality (0 – 3 months) in the multiple micronutrient group compared to iron-folate (RR = 0.82; 95% CI: 0.70 – 0.95; P = 0.010), with comparable results for post-neonatal mortality – from 29 days to 90 days after birth (RR = 0.70; 95% CI: 0.55 – 0.89). Our analysis of data from nine studies showed a non-significant effect on neonatal mortality (fixed effects: RR = 1.05; 95% CI: 0.92 – 1.19; random effects: RR = 1.17; 95% CI: 0.95 – 1.44). There was evidence that this risk might be related to the provision of skilled deliveries and standard of care in the health system. The 4 intervention trials conducted in populations where the majority of births occurred at home were associated with significant increase in the risk of neonatal mortality. However, this effect was not seen in the other 5 settings where the majority of births were in facilities. These results are generally consistent with a UNIMAPP trials analysis [43] with a notable, though non-significant increase in early neonatal mortality (OR = 1.23; 95% CI: 0.96 – 1.59), while there was a 6% non-significant reduction in late neonatal mortality (OR = 0.94; 95% CI: 0.73 – 1.23). These findings suggest that the use of multiple micronutrient supplements in populations to address maternal anemia and reduce the incidence of SGA, must be accompanied by the provision of skilled care at delivery and facility births to offset any potential increase in the risk of obstructed labour and birth asphyxia [44,45]. It must be noted that in most studies evaluated, despite reduction in rates of SGA, there was no reduction in neonatal mortality. A review of the relationship between IUGR and neonatal mortality largely based on observational studies did not distinguish if the effects on neonatal mortality were mediated through IUGR or other concurrent factors [5]. Applying the CHERG Rules for Evidence Review, we ranked the studies with neonatal mortality outcomes as high grade of evidence. Included studies had some minor limitations, especially insufficient power to detect differences in mortality between the groups. The studies, in general, were representative of the low-income populations. However, caution must be exercised in adapting the findings of multiple micronutrient supplements to programs without further effectiveness trials and robust evaluation in health systems.

It is surprising to note that the participants remained anemic in the included studies, even though they were taking seemingly adequate amounts of supplemental iron. There is no exact reason for this but this has been seen in many effectiveness trials over the years [40]. An important aspect to consider is the timing of initiation of multiple micronutrient supplements. Starting the supplementation during pregnancy may be too late for many women, especially those with pre-existing anemia [40]. Berger et al. from Vietnam had reported that the pre-pregnancy use of weekly iron–folic acid is associated with better iron status in the first and second trimesters and with reduced prevalence of LBW compared with pregnant women who only received daily iron–folic acid supplementation during pregnancy [46]. Another reason could be the amount of iron in the supplement, as higher dosages may be associated with more side effects of diarrhea and thus less absorption [40,47]. Similarly one possible reason could be infections, since these were not systematically treated across the trials. World health organization recommends the treatment of infections (especially deworming) for control of anemia along with supplementation of iron [48].

The observed benefit of multiple micronutrients in the SUMMIT trial [21] on early infant and post-neonatal mortality opens the interesting possibility that such intrauterine supplements may benefit infants beyond the neonatal period. This is further corroborated by the improved growth beyond infancy noted among recipients of maternal micronutrient supplements in Nepal [49]. Given the multiple effects of micronutrients in the regulation of body metabolism and growth [21], these effects are biologically plausible but need further evaluation with a range of additional outcomes, including child development indicators.

While there have also been calls for greater evaluation of potential interactions between various micronutrients [50], we feel that the field is ripe for well conducted effectiveness trials in health systems. The overall results of maternal micronutrient supplements call for further carefully conducted demonstration projects in health systems with adequate monitoring of pregnancy outcomes. Such studies may also overcome several of the limitations of size and power in the currently available literature. It must be underscored that intake of multivitamins and micronutrients are almost universal during pregnancy in developed countries and among the rich in developing countries [51]. Policy makers in health systems with adequate provision of skilled care may choose to introduce multiple micronutrient supplements in lieu of routine iron-folate for pregnant women with adequate monitoring and evaluation.

## Competing interests

We do not have any financial or non-financial competing interests for this review.

## Authors' contributions

Professor Zulfiqar A Bhutta developed the parameters for the review and secured support. Dr Batool Azra Haider and Dr Mohammad Yawar Yakoob undertook the literature search, data extraction and wrote the manuscript under Professor Bhutta’s supervision.

## Supplementary Material

Additional File 1 Excel Database of Extracted Data Click here for file

Additional File 2 Baseline characteristics of the included studies Click here for file

Additional File 3 Risk of bias for the included studies according to the latest recommendations of the Cochrane Handbook Click here for file

Additional File 4 **Effect of maternal multiple micronutrient supplementation versus iron folate on maternal anemia in the third trimester** A) Fixed model, B) Random model Click here for file

Additional File 5 **Effect of multiple micronutrients during pregnancy versus iron-folate on SGA babies with sub-group analysis according to maternal mean body mass index** A) Fixed model, B) Random model Click here for file

Additional File 6 **Effect of multiple micronutrient supplementation in pregnancy on neonatal mortality versus iron folate with sub-group analysis with respect to percentage of facility based births** A) Fixed model,B) Random model Click here for file
