# Supplementary material for: Effect of multiple micronutrient supplementation during pregnancy on maternal and birth outcomes
Source: BMC Public Health. 2011 Apr 13;11(Suppl 3):S19. doi: 10.1186/1471-2458-11-S3-S19 (PMC3231892; doi:10.1186/1471-2458-11-S3-S19)
Supplement: Additional File 4 — Effect of maternal multiple micronutrient supplementation versus iron folate on maternal anemia in the third trimester A) Fixed model, B) Random model [file 1471-2458-11-S3-S19-S4.docx]

**Additional File 4A: Effect of maternal multiple micronutrient supplementation versus iron folate on maternal anemia in the third trimester - Fixed model**

**Additional File 4B: Effect of maternal multiple micronutrient supplementation versus iron folate on maternal anemia in the third trimester - Random model**
