# Supplementary material for: Effect of multiple micronutrient supplementation during pregnancy on maternal and birth outcomes
Source: BMC Public Health. 2011 Apr 13;11(Suppl 3):S19. doi: 10.1186/1471-2458-11-S3-S19 (PMC3231892; doi:10.1186/1471-2458-11-S3-S19)
Supplement: Additional File 5 — Effect of multiple micronutrients during pregnancy versus iron-folate on SGA babies with sub-group analysis according to maternal mean body mass index A) Fixed model, B) Random model [file 1471-2458-11-S3-S19-S5.docx]

**Additional File 5A: Effect of multiple micronutrients during pregnancy versus iron-folate on SGA babies with sub-group analysis according to maternal mean body mass index - Fixed model**

**Additional File 5B: Effect of multiple micronutrients during pregnancy versus iron-folate on SGA babies with sub-group analysis according to maternal mean body mass index - Random model**
