# Supplementary material for: Effect of multiple micronutrient supplementation during pregnancy on maternal and birth outcomes
Source: BMC Public Health. 2011 Apr 13;11(Suppl 3):S19. doi: 10.1186/1471-2458-11-S3-S19 (PMC3231892; doi:10.1186/1471-2458-11-S3-S19)
Supplement: Additional File 6 — Effect of multiple micronutrient supplementation in pregnancy on neonatal mortality versus iron folate with sub-group analysis with respect to percentage of facility based births A) Fixed model,B) Random model [file 1471-2458-11-S3-S19-S6.docx]

**Additional File 6A: Effect of multiple micronutrient supplementation in pregnancy on neonatal mortality versus iron folate with sub-group analysis with respect to percentage of facility based births - Fixed model**

**Additional File 6B: Effect of multiple micronutrient supplementation in pregnancy on neonatal mortality versus iron folate with sub-group analysis with respect to percentage of facility based births - Random model**
